# Ribosome Biogenesis Regulator 1 Homolog (RRS1) Promotes Cisplatin Resistance by Regulating AEG-1 Abundance in Breast Cancer Cells

**DOI:** 10.3390/molecules28072939

**Published:** 2023-03-25

**Authors:** Junying Song, Cuixiu Peng, Runze Wang, Yanan Hua, Qinglan Wu, Lin Deng, Yi Cao, Li Zhang, Lin Hou

**Affiliations:** 1 Department of Biochemistry and Molecular Biology, School of Basic Medicine, Qingdao University, No. 308 Ningxia Road, Qingdao 266011, China; 2 Juxian No. 3 Middle School, No. 523 Chengyang South Road, Rizhao 267500, China; 3 Chongqing Key Laboratory of Sichuan-Chongqing Co-Construction for Diagnosis and Treatment of Infectious Diseases Integrated Traditional Chinese and Western Medicine, College of Medical Technology, Chengdu University of Traditional Chinese Medicine, Chengdu 611137, China; 4 Experimental Center for Undergraduates of Pharmacy, School of Pharmacy, Qingdao University, No. 308 Ningxia Road, Qingdao 266011, China

**Keywords:** RRS1, cisplatin resistance, AEG-1, interaction, breast cancer

## Abstract

Many ribosomal proteins are highly expressed in tumors and are closely related to their diagnosis, prognosis and pathological characteristics. However, few studies are available on the correlation between ribosomal proteins and chemoresistance. RRS1 (human regulator of ribosome synthesis 1), a critical nuclear protein involved in ribosome biogenesis, also plays a key role in the genesis and development of breast cancer by protecting cancer cells from apoptosis. Given that apoptosis resistance is one of the causes of the cisplatin resistance of tumor cells, our aim was to determine the relationship between RRS1 and cisplatin resistance in breast cancer cells. Here, we report that RRS1 is associated with cisplatin resistance in breast cancer cells. RRS1 silencing increased the sensitivity of MCF-7/DDP cells to cisplatin and inhibited cancer cell proliferation by blocking cell cycle distribution and enhancing apoptosis. AEG-1 (astrocyte elevated gene-1) promotes drug resistance by interfering with the ubiquitination and proteasomal degradation of MDR1 (multidrug resistance gene 1), thereby enhancing drug efflux. We found that RRS1 binds to and stabilizes AEG-1 by inhibiting ubiquitination and subsequent proteasomal degradation, which then promotes drug efflux by upregulating MDR1. Furthermore, RRS1 also induces apoptosis resistance in breast cancer cells through the ERK/Bcl-2/BAX signaling pathway. Our study is the first to show that RRS1 sensitizes breast cancer cells to cisplatin by binding to AEG-1, and it provides a theoretical basis to improve the efficacy of cisplatin-based chemotherapy.

## 1. Introduction

Breast cancer overlook lung cancer as the most frequently diagnosed cancer worldwide in 2020, accounting for nearly 12% of new cases [1]. Breast cancer cells show metabolic abnormalities and mitochondrial oxidative stress, and various tumor marker proteins are increased [2,3,4]. A combination of preoperative chemotherapy and surgery is the current treatment strategy for breast cancer patients. Because surgery affects the quality of life, chemotherapy is preferred for the early stages of breast cancer. Although cisplatin (DDP) is a broad-spectrum chemotherapeutic agent that has been used to treat various human malignancies [5], both intrinsic and acquired resistance greatly limit its clinical application [6,7]. Therefore, it is essential to explore the molecular mechanisms underlying chemoresistance in breast cancer in order to identify novel therapeutic targets.

Ribosomes are cellular protein factories, and their biogenesis is related to cell proliferation, protein secretion and renewal. The increased demand for ribosomes in cancer cells leads to abnormal ribosome synthesis, which is accompanied by an aberrant expression of ribosomal proteins and may affect tumorigenesis and drug response. Human regulator of ribosome synthesis 1 (RRS1) was discovered by Tsuno et al. in yeast in 1999. It regulates the maturation of 25S rRNA and the assembly of the 60S ribosomal subunit during ribosomal biosynthesis [8]. Recent studies show that RRS1 is aberrantly expressed in breast cancer [9,10,11], colorectal cancer [12], hepatocellular carcinoma [13] and thyroid cancer [14] and that is correlated to the proliferation and apoptosis of cancer cells [10]. Because apoptosis inhibition is one of the causes of cisplatin resistance [15,16], exploring the relationship between RRS1 and cisplatin resistance in breast cancer cells may determine the potential of ribosomal proteins as therapeutic targets.

AEG-1 was initially identified in primary human fetal brain glial cells, and it was found to be highly expressed in various tumors [17,18,19,20]. The activation of AEG-1 in breast cancer cells promotes drug resistance and metastasis [21] and desensitizes hepatoma cells to adriamycin by blocking the ubiquitination and proteasomal degradation of MDR1 [22]. AEG-1 also activates the pro-survival MEK/ERK, AKT, NF-κB and WNT signaling pathways in cancer cells [23]. Although studies show that RRS1 and AEG-1 interact in the endoplasmic reticulum to regulate the development of Huntington’s disease [24,25], there is no evidence of their interaction in the context of cisplatin resistance. The aim of this study was to determine whether RRS1 and AEG-1 interact to mediate cisplatin resistance in breast cancer cells.

To this end, we evaluated RRS1 expression levels in a cisplatin-resistant (MCF-7/DDP) and its parental cisplatin-sensitive (MCF-7) breast cancer cell line. RRS1 silencing significantly attenuated the malignant phenotype of the MCF-7/DDP cells and also sensitized the cells to cisplatin. Furthermore, RRS1 increased the content of AEG-1 protein by inhibiting its ubiquitination and proteasomal degradation, and AEG-1 promoted drug efflux by upregulating MDR1. In addition, AEG-1 also induced apoptosis resistance through the ERK/Bcl-2/BAX signaling pathway. Thus, both mechanisms synergistically promoted the cisplatin resistance of breast cancer cells. Our findings provide a theoretical basis for the treatment of breast cancer and improving the efficacy of cisplatin chemotherapy.

## 2. Results

### 2.1. RRS1 Was Highly Expressed in Cisplatin-Resistant Breast Cancer Cells and Regulated Chemosensitivity

**Figure 1:**
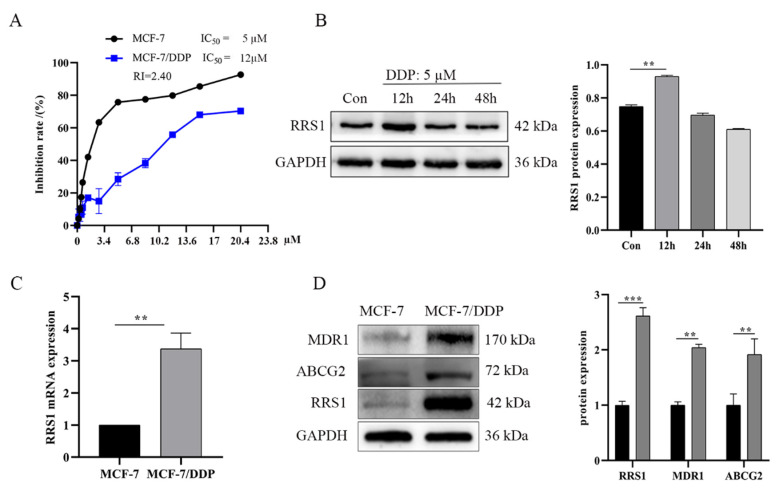
Identification of the drug resistant cell MCF-7/DDP cell line and RRS1 expression. (**A**) IC_50_ of DDP for MCF-7 and MCF-7/DDP cells. (**B**) MCF-7 cells were treated with it IC_50_ of cisplatin at 12 h, 24 h and 48 h; RRS1 protein expression was measured. (**C**) RRS1 mRNA levels in the indicated cell lines. (**D**) Immunoblot showing RRS1, ABCG2 and MDR1 protein levels in the indicated cell lines. Data are expressed as mean ± SD of at least three independent experiments. ** *p* < 0.01, *** *p* < 0.001. RRS1, human regulator of ribosome synthesis 1; ABCG2, ATP-binding cassette sub-family G member 2; MDR1, multidrug resistance protein 1.

The half maximal inhibitory concentrations (IC_50_) of cisplatin (DDP) for MCF-7 and MCF-7/DDP cells were 5µM and 12µM, respectively, after 24 h of cisplatin exposure (Figure 1A). MDR1 (multidrug resistant gene 1, MDR1/P-gp) and ABCG2 (Breast cancer resistance protein, BCRP/ABCG2) pump out drugs from cells in an ATP-dependent manner [26]. We found that MDR1 and ABCG2 were overexpressed in MCF-7/DDP cells compared to the MCF-7 cells. The 2.4-fold higher IC_50_ and the higher MDR1 and ABCG2 expression in MCF-7/DDP cells are consistent with cisplatin resistance. Subsequently, the MCF-7 cells were treated with the corresponding IC_50_ of cisplatin. As shown in Figure 1B, the expression of RRS1 increased significantly in MCF-7 cells after 12 h of cisplatin treatment, suggesting that cisplatin can induce RRS1 expression in breast cancer cells. In addition, RRS1 protein and mRNA levels were significantly higher in the MCF-7/DDP cells compared to those in the parental MCF-7 cells (Figure 1C,D). These findings suggest that RRS1 is associated with the sensitivity of breast cancer cells to cisplatin.

### 2.2. RRS1 Knockdown Reduced the Proliferative Rates of MCF-7/DDP Cells and Sensitized Them to Cisplatin

**Figure 2:**
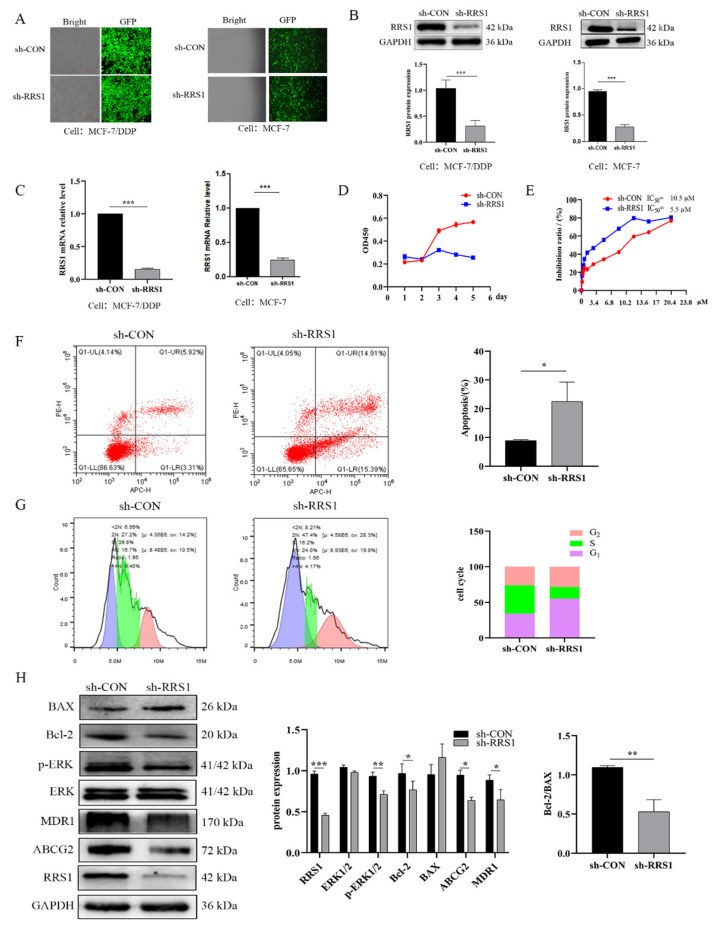
RRS1 knockdown reduced the proliferative rates of MCF-7/DDP cells and sensitized them to cisplatin. (**A**) MCF-7/DDP and MCF-7 cells were infected with lentiviruses, and transduction efficiency after 72 h was determined in terms of GFP fluorescence. Scale bars = 100 µm. (**B**) Immunoblot showing RRS1 protein levels in the MCF-7/DDP and MCF-7 cells infected with the RRS1-shRNA or scrambled shRNA lentiviruses. (**C**) RRS1 mRNA levels in the MCF-7/DDP and MCF-7 cells treated. (**D**) Proliferation rates of sh-CON and sh-RRS1 MCF-7/DDP cells. (**E**) IC_50_ of DDP for control and RRS1-knockdown MCF-7/DDP cells. (**F**) Flow cytometry plots showing apoptosis rates in the indicated cells. (**G**) Cell cycle distribution of the indicated cells. Purple bars, percentage of cells in G1 phase; green bars, percentage of cells in S phase; pink bars, percentage of cells in G2 phase. Data are the means of three experiments. (**H**) Immunoblots showing the expression levels of the indicated proteins in the sh-CON and sh-RRS1 groups’ cells. Data are the means ± SDs of at least three independent experiments. * *p* < 0.05, ** *p* < 0.01, *** *p* < 0.001. RRS1, human regulator of ribosome synthesis 1; DDP, cisplatin; GFP, green fluorescent protein.

To further detect the role of RRS1 in breast cancer cells’ cisplatin resistance, we silenced the RRS1 gene using specific shRNA, which significantly decreased both mRNA and protein expression (Figure 2A–C). In MCF-7 cells, the knockdown efficiency of lentivirus was not affected by cisplatin resistance, and knockdown efficiency reached more than 70%. Depletion of RRS1 in the resistant cells significantly increased their sensitivity to cisplatin. As shown in Figure 2E, the IC50 values of cisplatin decreased from 10.5 µM in the control MCF-7/DDP cells to 5.5 µM in the RRS1-knockdown counterparts (sh-RRS1), a level close to those of MCF-7 cells. The expression levels of ABCG2 and MDR1 were significantly higher in the MCF-7/DDP cells compared to those in MCF-7 cells, which was consistent with drug resistance. However, RRS1 knockdown downregulated both transporter proteins (Figure 2H), which led to lower drug efflux and increased drug accumulation in the cells.

Knocking down RRS1 also markedly reduced the proliferative rates of MCF-7/DDP cells compared to the control cells transfected with sh-CON (Figure 2D). Consistent with this, RRS1 knockdown blocked the cell cycle at G_1_ phase (Figure 2G), and also significantly increased the apoptosis rates of the drug-resistant cell line (Figure 2F). Furthermore, the pro-proliferative p-ERK and the anti-apoptotic protein Bcl-2 decreased significantly after RRS1 knockdown. In contrast, the pro-apoptotic protein BAX was upregulated in the RRS1-knockdown cells, and the Bcl-2/BAX ratio was decreased (Figure 2H), results which were consistent with changes in cell cycle and apoptosis status. These findings indicate that RRS1 modulated cisplatin resistance by increasing proliferative activity and inhibiting apoptosis.

### 2.3. AEG-1 Might Participate in Cisplatin Resistance Mediated by RRS1

**Figure 3:**
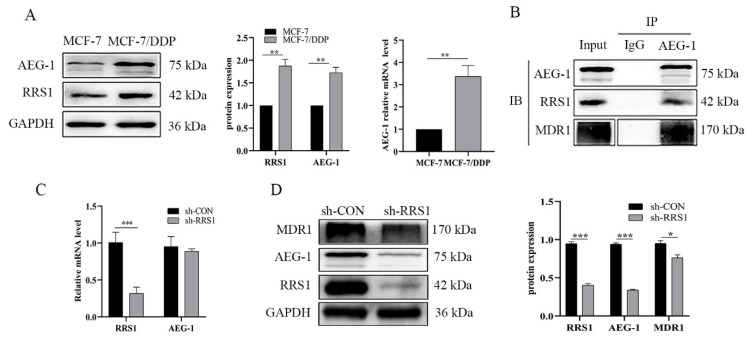
AEG-1 might participate in cisplatin resistance mediated by RRS1. (**A**) The expression of RRS1 and AEG-1 proteins in MCF-7 and MCF-7/DDP cells. (**B**) The interaction between AEG-1, RRS1 and MDR1 proteins in MCF-7/DDP cells. Cell lysates were subjected to immunoprecipitation with anti-AEG-1 antibodies, followed by immunoblotting with anti-AEG-1, anti-RRS1 and anti-MDR1 antibodies. (**C**) AEG-1 mRNA levels in sh-CON and sh-RRS1 MCF-7/DDP cells. (**D**) AEG-1 protein levels in sh-CON and sh-RRS1 MCF-7/DDP cells. Data are expressed as means ± SDs of at least three independent experiments. * *p* < 0.05, ** *p* < 0.01, *** *p* < 0.001. RRS1, human regulator of ribosome synthesis 1; DDP, cisplatin; AEG-1, astrocyte elevated gene-1; MDR1, multidrug resistance protein 1.

AEG-1 is known to confer resistance to broad-spectrum chemotherapeutics, including adriamycin, cisplatin, paclitaxel, hydrogen peroxide and 4-hydroxycyclophosphamide [27]. Studies show that AEG-1 blocked the ubiquitination and proteasomal degradation of MDR1 without affecting its transcription levels [22]. Although there is evidence of direct interaction between RRS1 and AEG-1 in the endoplasmic reticulum to regulate the development of Huntington’s disease [24,25], it is unclear whether the interaction between these two proteins is involved in cisplatin resistance as well. We found that the expression levels of both RRS1 and AEG-1 were significantly higher in the MCF-7/DDP cells compared to the MCF-7 cells (Figure 3A). Furthermore, we confirmed the direct interaction of AEG-1 with RRS1 and MDR1 via Co-IP (Figure 3B), although there were no interactions between RRS1 and MDR1. In addition, RRS1 silencing did not lead to any evident changes in AEG-1 mRNA levels (Figure 3C) but significantly reduced protein content (Figure 3D). Taken together, RRS1 increased AEG-1 protein levels in breast cancer cells without affecting their transcriptional activity.

### 2.4. RRS1 Blocked AEG-1 Ubiquitination and Proteasome Degradation

**Figure 4:**
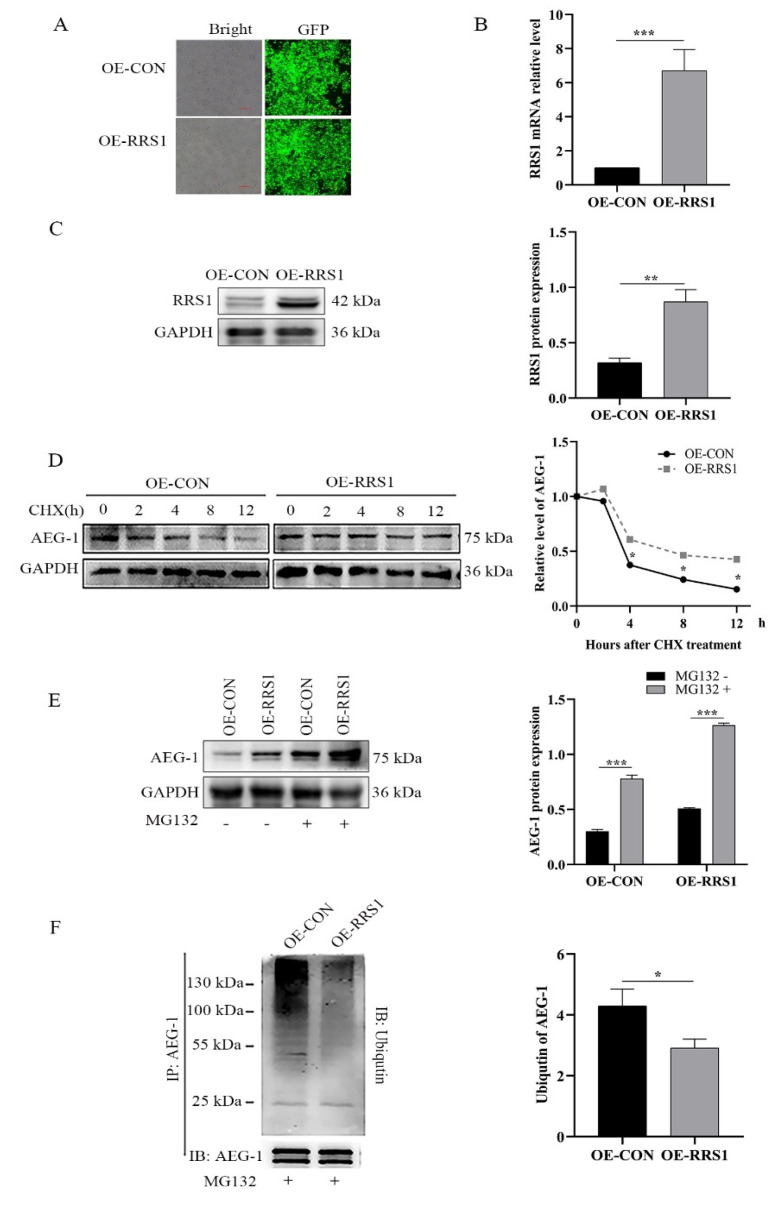
RRS1 blocked AEG-1 ubiquitination and proteasomal degradation. (**A**) A stable transgenic strain with RRS1 highly expressed was constructed. MCF-7/DDP cells were infected with lentivirus, and the fluorescence transfection efficiency was detected after 72 h, with scale = 100 µm. After 72 h of infection, the cells were screened with puromycin of 1 µg/mL for 1 week. (**B**) RRS1 mRNA levels in OE-CON and OE-RRS1 MCF-7/DDP cells. (**C**) Immunoblot showing RRS1 protein levels in the OE-CON and OE-RRS1 MCF-7/DDP cells. (**D**) The half-life of AEG-1 protein in cells treated with CHX (710 µM). (**E**) AEG-1 protein levels in cells treated with MG132 (10 µM) for 12 h. (**F**) Cells were treated with MG132 (10 µM) for 12 h; the cell lysates were subjected to immunoprecipitation with anti-AEG-1 antibodies, followed by western blotting with anti-ubiqutin antibodies. Data are expressed as means ± SDs of at least three independent experiments. * *p* < 0.05, ** *p* < 0.01, *** *p* < 0.001. RRS1, human regulator of ribosome synthesis 1; AEG-1, astrocyte elevated gene- 1; CHX, cycloheximide.

We further studied whether RRS1 could increase AEG-1 content in breast cancer drug-resistant cells by regulating protein stability. Protein stability was detected by using protein synthesis inhibitor CHX. Because AEG-1 decreased obviously after RRS1 knockdown, and because the cells treated with a high concentration of CHX would seriously interfere with protein synthesis in a short time, we established an RRS1-overexpressing cell line in order to stabilize protein levels (Figure 4A), and we treated the cells with the protein synthesis inhibitor CHX. As shown in Figure 4B, overexpressing RRS1 markedly extended the half-life of AEG-1 protein even in the absence of protein synthesis. In addition, the proteasome inhibitor MG132 augmented the increase in AEG-1 protein levels (Figure 4C). To determine whether RRS1 regulates the ubiquitination of AEG-1protein, we treated the OE-CON and OE-RRS1 cells with MG132. The cell lysates were subjected to immunoprecipitation with anti-AEG-1 antibodies, and then the immunoprecipitated lysates were subjected to Western blot analysis using anti-ubiqutin antibodies. Compared to the OE-CON cells, the levels of ubiquitinated AEG-1 decreased significantly in the OE-RRS1 cells (Figure 4D). Taken together, RRS1 upregulated AEG-1 protein levels in the MCF-7/DDP cells by blocking its ubiquitination and proteasomal degradation.

### 2.5. Cisplatin Induced RRS1 Overexpression In Vivo

**Figure 5:**
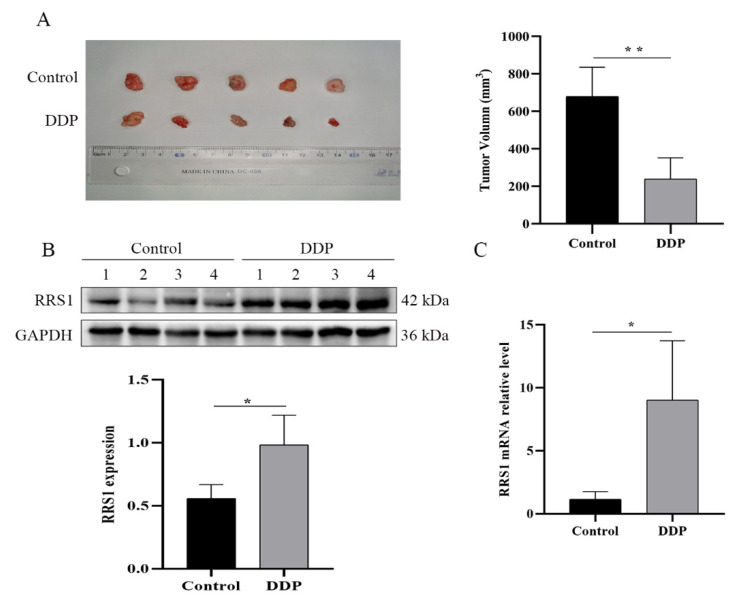
Cisplatin induced RRS1 overexpression in vivo. MCF-7 cells were injected into nude mice, which were injected with 15 mg/kg DDP once weekly for 4 weeks. (**A**) Representative images of tumors extracted from the indicated groups. (**B**) Immunoblot showing RRS1 protein levels in the tumor tissues of the indicated groups. (**C**) RRS1 mRNA levels in the indicated tumor groups. Data are expressed as means ± SDs of at least three independent experiments. * *p* < 0.05, ** *p* < 0.01. DDP, cisplatin.

To further validate the correlation between RRS1 and cisplatin treatment, MCF-7 cells were injected subcutaneously into athymic nude mice to generate breast tumor models. As shown in Figure 5A, the tumors were significantly smaller in the cisplatin-treated vs. the untreated control mice, which coincided with increased levels of RRS1 mRNA and protein in the cisplatin-treated tumors (Figure 5B,C). These results in vivo further confirm that RRS1 is related to the cisplatin resistance of breast cancer cells.

## 3. Materials and Methods

### 3.1. Cell Line Authentication

The MCF-7/DDP cell line was purchased from Chuanqiu Biology (Shanghai, China) in October 2019, and it was tested and authenticated by STR in June 2018. Genomic DNA was extracted from the MCF-7/DDP cells and amplified using the Powerplex^TM^16 ID System STR compound amplification kit (Promega, Madison, WI, USA). An ABI 3130xl genetic analyzer(ABI, Foster, CA, USA) was used to detect the STR locus and sex gene amelogenin, and the genomic map and genotype were analyzed. The MCF-7 cell line was obtained from the Laboratory of Affiliated Hospital of Qingdao University in October 2016 and tested in August 2020. Genomic DNA was extracted using the Microread Genomic DNA kit (Suzhou Microread Genetics, Beijing, China), and 20 STR sites and sex loci were amplified using the Microreader^TM^21 ID System (Suzhou Microread Genetics, Beijing, China). The PCR products were detected using an ABI 3730xl genetic analyzer and GeneMapperID-X (ABI, Foster, CA, USA), and they were compared to ATCC and DSMZ databases.

### 3.2. Cell Culture and Chemical Reagents

MCF-7 cells were cultured in Dulbecco’s modified Eagle medium (DMEM; HyClone, Logan, UT, USA) and supplemented with 10% fetal bovine serum (FBS; ExCell Bio, Shanghai, China). The MCF-7/DDP cells were cultured in RPMI-1640 (HyClone, Logan, UT, USA) supplemented with 10% FBS (ExCell Bio, Shanghai, China) and 0.5 µM DDP (Solarbio, Beijing, China) at 37 °C under 5% CO_2_. Total RNA and proteins were extracted from the cells in the logarithmic phase of growth for molecular assays. Depending on the experiment, the cells were incubated in the presence of one or more of 2.1 µM puromycin (Yeasen, Shanghai, China), 710 µM cycloheximide (CHX) and 10 µM MG132 (Selleck, Shanghai, China).

### 3.3. Lentivirus Transfection

Cells in the logarithmic growth phase and 80–90% confluency were harvested, resuspended in complete medium and seeded in 6-well plates at the density of 2 × 10^5^ cells per well. The shRNA targeting RRS1 (sh-RRS1), control shRNA (sh-CON), overexpressed RRS1 (OE-RRS1) and scrambled control (OE-CON) were assembled using pSuper constitutive expression constructs (Genecard, China), and the cells were infected with their respective lentiviruses at an MOI of 20. The medium was changed 12 h after infection, and the efficiency of transduction was observed 48–72 h later under a fluorescence microscope. Total protein and RNA were extracted by following standard protocols.

### 3.4. Quantitative Reverse Transcription PCR (RT-qPCR)

Total RNA was extracted from cultured cells or tumors using a Trizol RNA isolation kit (Vazyme, Nanjing, China) and amplified by performing the RT-qPCR according to the manufacturer’s protocol (Vazyme, Nanjing, China). All samples were analyzed in triplicate. The primer sequences were as follows: GAPDH forward 5′-AGAAGGCTGGGGCTCATTTG-3′ and reverse 5′-AGGGGCCATCCACAGTCTTC-3′; RRS1 forward 5′-CCCTACCGGACACCAGAGTAA-3′ and reverse 5′-CCGAAAAGGGGTTGAAACTTCC-3; AEG-1 forward 5′-CGAGAAGCCCAAACCAAATG-3′ and reverse 5′-TGGTGGCTGCTTTGCTGTT-3′.

### 3.5. Cisplatin Sensitivity Analysis

The MCF-7/DDP cells were seeded in 96-well plates at a density of 3 × 10^3^ cells per well and cultured until the logarithmic phase. DDP was added to the final concentrations of 0, 0.2, 0.3, 0.6, 1.3, 2.6, 5, 8, 12, 15 and 20 µM. After 24 h of incubation, 10 µL CCK-8 solution (Solarbio, Beijing, China) was added to each well. The absorbance at 450 nm was measured, and the inhibition ratios and IC_50_ were calculated.

### 3.6. Cell Proliferation Assay

The suitably treated cells were seeded in 96-well plates at the density of 3 × 10^3^ cells per well, and 10 µL CCK-8 solution (Solarbio, Beijing, China) was added to each well after varying durations. The cells were incubated for another 2 h, and the absorbance at 450 nm was measured.

### 3.7. Cell Cycle and Apoptosis

Cell cycle distribution was detected with 25 µg propidium iodide (Solarbio, Beijing, China). Briefly, the MCF-7/DDP cells were seeded in 6-well plates at the density of 2 × 10^5^ cells per well and cultured for 12 h; then, the cells were transfected with control and RRS1 shRNA lentivirus for 48 h. The cells were collected using trypsin without EDTA and placed into a 15 mL centrifuge tube, into which 1.5 mL cool PBS and 3.5 mL absolute ethanol were added, and the mixture was fixed at 4 °C overnight. The mixture was then centrifuged, and the supernatant was discarded. The precipitate was added into 200 μL PBS and 0.5 mg RNase and incubated at 37 °C for 30 min; then, 25 µg PI solution was added, and the mixture was incubated at room temperature in the dark for 30 min. The stained cells were analyzed using a Beckman flow cytometer. 

### 3.8. TUNEL Staining

Apoptosis was detected using an Annexin V-APC/PI apoptosis detection kit (Elabscience, Wuhan, China). The cells transfected with the sh-CON, and sh-RRS1 lentivirus were harvested and then incubated with 500 µL binding buffer, 2.5 μL Annexin V-APC and 2.5 μL PI reagent for 15 min at room temperature in the dark. The stained cells were analyzed using a Beckman flow cytometer.

### 3.9. Co-Immunoprecipitation (Co-IP)

Co-IP was performed using a protein A/G kit (Epizyme, Shanghai, China). The beads were activated via overnight incubation in 25 µL of lysis buffer. The suitably treated MCF-7/DDP cells were homogenized in cell lysis buffer at 4 °C for 30 min, and protein concentrations were measured using the BCA assay (Solarbio, Beijing, China). Around 500 µL of each lysate was incubated overnight with the anti-AEG-1 antibody (1 µg, Abcam, ab227981) or with IgG (1 µg, Bioss, Beijing, China, bs-0297R) in the presence of activated beads. The resulting precipitate was washed with lysis buffer, separated via SDS-PAGE and probed using the indicated antibodies with immunoblotting.

### 3.10. Protein Stability Assay

OE-CON and OE-RRS1 cells of 80% confluency were treated with 710 µM CHX, and protein was extracted at the indicated time points and analyzed via Western blotting.

### 3.11. Xenograft Assay

Four-weeks-old male nude (BALB/c) mice (VitalRiver, China) were injected subcutaneously with MCF-7 cells (5 × 10^6^ cells/0.2 mL per mouse, medium/matrix = 1). Once the xenografts approximately measured 100 mm^3^ (around 1 week later), the mice were randomly divided into control (100 µL normal saline) and DDP (15 mg/kg) groups (*n* = 5 each) and injected intraperitoneally with their respective reagents once every week for four weeks. The longest (a) and shortest (b) diameters of the tumors were measured using digital calipers, and the tumor volume (V) was calculated as a × b^2^/2. The mice were then euthanized, and the tumors were harvested for further analysis.

### 3.12. Western Blotting

The cells were lysed in RIPA buffer (Sparkjade, Jinan, China) supplemented with protease inhibitor cocktail (Servicebio, Wuhan, China) and phosphatase inhibitors A and B on ice for 30 min. The lysates were centrifuged for 15 min at 12,000 rpm, and their protein content was measured. Equal amounts of protein per sample were resolved on 10% SDS-PAGE gels and transferred to a PVDF membrane. After blocking with 5% BSA in TBST for 2 h at room temperature, the membranes were incubated overnight with antibodies targeting GAPDH (Abclonal, Wuhan, China, AC002), RRS1 (Abcam, Cambridge, UK, AB188161), AEG-1 (Abcam, Cambridge, UK, ab227981), ABCG2 (ZenBio, Research Triangle Park, NC, USA, R26465), MDR1 (Proteintech, Wuhan, China, 22336-1-AP), ERK (ZenBio, NC, USA, 340373), p-ERK (ZenBio, NC, USA, 340767), BAX (CST, Danvers, MA, USA, #89477), Bcl-2 (CST, Danvers, MA, USA, #3498), BAD (Abcam, Cambridge, UK, ab32445), p-BAD (Abcam, Cambridge, UK, ab129192) and ubiquitin (Proteintech, Wuhan, China, 10201-2-AP) at 4 °C. The membranes were washed thrice with TBST and then incubated with HRP-conjugated secondary antibody (1:1000; Bioss, Beijing, China, bs-0295G-HRP), followed by three more washes with TBST. The positive bands were visualized via ECL (MDBio, Taiwan, China).

### 3.13. Statistical Analysis

The data was analyzed using the SPSS 13.0 program. All data are expressed as means ± standard deviations (SD), and Student’s *t*-test was used to compare the differences between the two groups. *p* < 0.05 was considered to be statistically significant.

## 4. Discussion

Breast cancer is a commonly diagnosed cancer in women, with a global incidence rate of 24.5 % [1]. Although chemotherapy is still the first line of treatment for early breast cancer, its clinical outcomes are suboptimal due to drug resistance. In fact, acquired drug resistance is the major cause of treatment failure and mortality in breast cancer patients because it is accompanied by increased viability and metastatic potential in the tumor cells [28]. Therefore, it is critical to investigate the molecular mechanisms underlying chemoresistance.

Studies show that the malignant transformation of cells is often accompanied by increased ribosomal biogenesis, which, in turn, is the result of the aberrant overexpression of oncogenes and the inhibition of tumor suppressors [29]. RRS1 is one of approximately 70 proteins that interact with rRNAs in large and small ribosomal subunits. During ribosome biosynthesis, RRS1 combines with ribosomal product factor 2 (RPF2) and recruits RPL5, RPL11 and 5S rRNA to the pre-90s subunit, which is processed into the mature 60S subunit [30,31,32]. There is evidence indicating that ribosomal proteins also regulate the multidrug resistance of tumor cells [33]. A comparison of the gene profiles of the cisplatin-resistant HNSC cell line UMSCC10b/Pt-S15 and the parental UMSCC10b cells revealed that the RPS28 and EF-1α were upregulated in cisplatin-resistant cells. In addition, patients with high expression of phosphorylated RPS6 had better prognoses, and a lack of upregulation might indicate drug resistance [34]. We confirmed that RRS1 promotes the proliferation of breast cancer cells and inhibits apoptosis [9], whereas apoptosis inhibition was one of the mechanisms of cisplatin resistance [15,16]. Therefore, we explored the effect of RRS1 on cisplatin resistance in breast cancer cells.

Cisplatin treatment upregulated RRS1 in breast cancer cells, and the subsequent knockdown of RRS1 reversed the drug resistance of MCF-7/DDP cells. Furthermore, RRS1 knockdown also affected the proliferation, cell cycle distribution and apoptosis of the MCF-7/DDP cells via the ERK signaling pathway and Bcl-2/BAX proteins. AEG-1 activation is known to promote drug resistance and metastasis in breast cancer cells [21]. It also promotes MDR1 translation by blocking ubiquitination and proteasomal degradation without affecting its transcription level [22]. We found that both RRS1 and AEG-1 were upregulated in the MCF-7/DDP cells compared to the MCF-7 cells and that knocking down RRS1 decreased AGE-1 levels. However, RRS1 knockdown did not affect AEG-1 mRNA expression, suggesting that RRS1 does not regulate AEG-1 at the transcriptional level. We were able to confirm interaction between RRS1 and AEG-1 proteins, and we found that RRS1 overexpression significantly extended the half-life of AEG-1 in MCF-7/DDP cells. In addition, the overexpression of RRS1 increased AEG-1 protein abundance by inhibiting its ubiquitination and proteasomal degradation. Thus, RRS1 increased AEG-1 levels inMCF-7/DDP cells by increasing its stability.

We only got some preliminary results on the role of RRS1 in breast cancer cisplatin resistance. Nevertheless, we were not able to prove any interaction between RRS1 and MDR1 proteins, which raises the possibility of other mechanisms through which RRS1 regulates MDR1. Further studies should focus on whether RRS1 interacts directly with AEG-1 and explore the specific mechanisms and ubiquitination sites.

**Figure 6:**
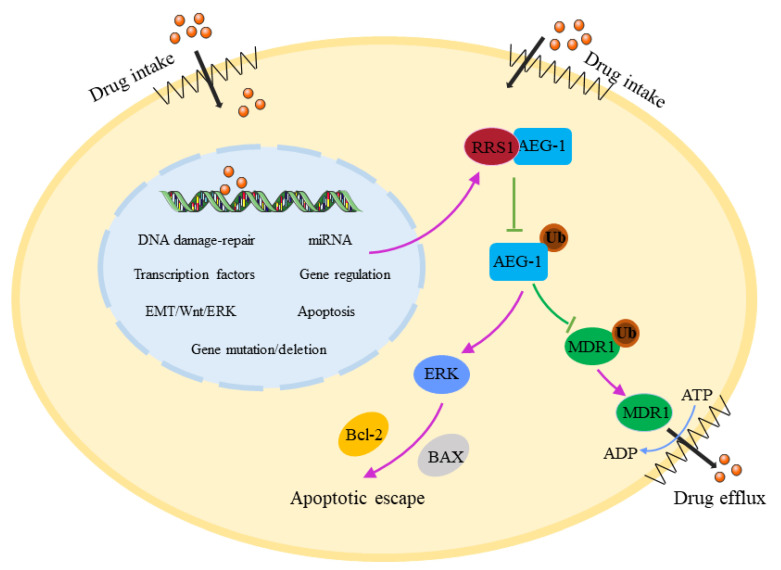
The mechanism of RRS1-regulated cisplatin resistance in breast cancer cells.

In conclusion, we show for the first time that RRS1 might regulate cisplatin resistance in breast cancer cells by inhibiting AEG-1 ubiquitination and proteasome degradation. Then, AEG-1 promotes drug efflux by upregulating MDR1, and it directly inhibits apoptosis through the ERK/Bcl-2/BAX signaling pathway. Both mechanisms synergistically promote cisplatin resistance in breast cancer cells (Figure 6). Our findings provide a theoretical basis for the treatment of breast cancer and for improving the efficacy of cisplatin chemotherapy.

## Data Availability

All data generated or analyzed during this study are included in this published article.
